# Roles of Neuropeptide Y in Neurodegenerative and Neuroimmune Diseases

**DOI:** 10.3389/fnins.2019.00869

**Published:** 2019-08-20

**Authors:** Chunrong Li, Xiujuan Wu, Shan Liu, Yue Zhao, Jie Zhu, Kangding Liu

**Affiliations:** ^1^Neuroscience Center, Department of Neurology, The First Hospital of Jilin University, Jilin University, Changchun, China; ^2^Department of Neurobiology, Care Sciences and Society, Karolinska Institute, Karolinska University Hospital Huddinge, Stockholm, Sweden

**Keywords:** neuropeptide Y, neurodegenerative diseases, Alzheimer’s disease, Parkinson’s disease, neuroimmune disorders, Guillain-Barré syndrome

## Abstract

Neuropeptide Y (NPY) is a neurotransmitter or neuromodulator that mainly exists in the nervous system. It plays a neuroprotective role in organisms and widely participates in the regulation of various physiological processes *in vivo*. Studies in both humans and animal models have been revealed that NPY levels are altered in some neurodegenerative and neuroimmune disorders. NPY plays various roles in these diseases, such as exerting a neuroprotective effect, increasing trophic support, decreasing excitotoxicity, regulating calcium homeostasis, and attenuating neuroinflammation. In this review, we will focus on the roles of NPY in the pathological mechanisms of neurodegenerative and neuroimmune diseases, highlighting NPY as a potential therapeutic target in these diseases.

## Introduction

Neuropeptide Y (NPY) is a polypeptide composed of 36 amino acid residues, and belongs to the NPY family of neuroendocrine peptides, which also includes peptide YY and pancreatic polypeptide. It was first purified from the porcine brain by Caroline Tatemoto, a Swedish scientist at the Karolinska Institute, in 1982 (Yi et al., 2018). The functions of NPY comprise the regulation of brain activity, stress coping, digestion, blood pressure, heart rate, body metabolism, and immune function (Thorsell and Mathe, 2017). NPY is widely distributed in the central and peripheral tissues of mammals, especially in the nervous system. A growing body of literature, both in humans and rodent models, has revealed that brain NPY levels are altered in some neurodegenerative and neuroimmune diseases. NPY also plays an important regulatory role in the immune function and inflammatory response of the central nervous system (CNS) such as modulation of chemotaxis of immune cells, phagocytosis, and production and release of cytokines (Ferreira et al., 2010). In this review, we elucidate the roles of NPY in the pathological mechanisms of neurodegenerative and neuroimmune diseases and highlight its potential possibility as a therapeutic target in these disorders.

## Npy and Npy Receptors

In the CNS, the highest concentrations of NPY are present in the hippocampus, produced mainly by γ-aminobutyric acid (GABA) ergic interneurons, which can inhibit the transmission of excitatory amino acids (Xapelli et al., 2006). NPY is also distributed in the cerebral cortex, hypothalamus, thalamus, brainstem, and cerebellum. NPY has been implicated in epilepsy, learning, memory, feeding, and endocrine secretions (Kovac and Walker, 2013; O’Loughlin et al., 2014). The different biological actions of NPY in the CNS are accomplished mainly through binding to and activating various NPY receptors in several brain regions. NPY receptors are G-protein-coupled receptors, five of which have already been cloned from mammals: the Y1, Y2, Y4, Y5, and Y6 receptors (Diaz-delCastillo et al., 2018). The distribution of each of these Y receptors in the brain is different. The messenger ribonucleic acids (mRNAs) of Y1 and Y2 receptors are expressed at high levels in brain regions involved in memory function, such as the hippocampus, amygdala, thalamus, hypothalamus, and cerebral cortex. The expression of Y4 receptors is limited to only a few brain regions, including the medial preoptic area, nucleus tractus solitarii, paraventricular nucleus of the hypothalamus, and area postrema. Y5 receptors are found in several limbic brain areas, including the hippocampus, cingulate cortex, and the thalamic and hypothalamic nuclei. Y6 receptors are only present in some mammals (Gao et al., 2017). Y6 receptors are non-functional in several mammals including humans, and their physiological functions are not known yet. Y1 and Y2 receptors are key receptors of NPY, and are involved in vasoconstriction. Y1, Y2, and Y5 receptors are essential for the regulation of animal feeding behavior, whereas Y1, Y2, and Y4 receptors are essential for the regulation of anxiety and depression in animals. Y1 receptors are closely related to the regulation of immune function. NPY affects cell migration, cytokine release and antibody production through its Y1 receptors (Wheway et al., 2007; Farzi et al., 2015; Schmitz et al., 2017). NPY produces neuroprotective effects via Y2 receptors by alleviating the excitatory neurotoxic effect of kainic acid on CA1 and CA3 pyramidal cells (Xapelli et al., 2008; Gonçalves et al., 2012). Wu and Li (2005) and Farzi et al. (2015) further found that intraventricular injection of Y2 and Y5 agonists of NPY could alleviate the apoptosis of vertebral neurons in CA1 and CA3 regions caused by kainic acid. The distributions and functions of NPY receptors are summarized in the Table 1.

**TABLE 1:** Distributions and effects of NPY in human brain.

**NPY receptors**	**Distributions of NPY receptors**	**Effects of NPY receptors**
Y1	hippocampus, amygdala, thalamus, hypothalamus, cerebral cortex	vasoconstriction, regulating feeding behavior, regulating anxiety and depression
Y2	hippocampus, amygdala, thalamus, hypothalamus, cerebral cortex	vasoconstriction, regulating feeding behavior, regulating anxiety and depression, neuroprotective effects
Y4	medial preoptic area, NTS, PVH, area postrema	regulating anxiety and depression
Y5	hippocampus, cingulate cortex, thalamic, hypothalamic nuclei	regulating feeding behavior, neuroprotective effects
Y6	only present in some mammals	

*NTS, nucleus tractus solitarii; PVH, paraventricular nucleus of the hypothalamus; NPY, Neuropeptide Y.*

## Roles of Npy in Neurodegenerative Diseases

Neurodegenerative diseases are characterized by late onset, progressive clinical course, and neuronal loss with regional specificity in the CNS, such as Alzheimer’s disease (AD), Huntington’s disease (HD), Parkinson’s disease (PD), and Machado–Joseph disease (MJD) (Nobrega et al., 2019). Alterations in NPY in these neurodegenerative diseases are summarized in Table 2.

**TABLE 2:** Alteration of NPY levels in neurodegenerative diseases.

**Diseases**	**Changes of NPY levels**	**Regions of NPY level changes**	**References**
AD	reduced neuropeptide Y-like immunoreactivity (NPYLI) content decreased NPY mRNAs expression decreased NPY plasma content	AD cerebral cortex hippocampal and cortical of transgenic mouse models plasmas of AD	Koide et al., 1995; Ramos et al. 2006; Chen X.Y. et al., 2019
PD	increased NPY mRNA expression	caudate nucleus, putamen and nucleus accumbens of PD	Cannizzaro et al., 2003
HD	increased NPY expression	basal ganglia cortex and the subventricular zone of HD	Dawbarn et al., 1985
MJD	decreased NPY level	cerebella of MJD	Duarte-Neves et al., 2015

*AD, Alzheimer’s disease; HD, Huntington’s disease; PD, Parkinson’s disease; MJD, Machado-Joseph disease; NPY, Neuropeptide Y.*

### NPY in Alzheimer’s Disease

Alzheimer’s disease is considered as the most common neurodegenerative disease characterized by impairments in learning and memory functions. Pathophysiologically, the hallmarks of AD include formation of amyloid β (Aβ) plaques, neurofibrillary tangles, loss of neurons and dysfunction of synapses, cerebral amyloid angioplasty, and granular-vacuolar degeneration (Lane et al., 2018). Although the pathogenesis of AD is poorly understood, several factors, including genetic factors, inflammation, free radicals, cholinergic alterations, metal ions, viruses, oxidative stress, and decline in estrogen levels are involved (Camandola and Mattson, 2015). Beal et al. (1986) found that neuropeptide Y-like immunoreactivity (NPYLI) reduced widely in the cerebral cortex of AD by comparing the concentrations of NPYLI in post-mortem brains of AD and healthy controls. The alterations of NPY in AD or animal models of AD were reported in succession (Chen X.Y. et al., 2019). Plasma NPY levels in patients with AD were significantly decreased. This finding was revealed by measuring the plasma content of NPY in AD patients and healthy controls (Koide et al., 1995). (Ramos et al., 2006) found that the expressions of NPY mRNAs was also decreased in the hippocampal and cortical regions of a transgenic (tg) mouse model of AD.

Sporadic AD is mainly caused by an imbalance between Aβ production and clearance, resulting in Aβ accumulation. Rose et al. (2009) elucidated the effect of NPY on Aβ in AD. NPY C-terminal fragments (CTFs) were cleaved from full-length NPY by extracellular endopeptidase neprilysin. This generated a prominent CTF comprising amino acids 21–36 and 31–36. Infusion of these NPY CTFs into the brains of mouse models of AD ameliorated the neurodegenerative pathology (Rose et al., 2009). In addition, the amidated NPY CTFs protected human neuronal cultures from the neurotoxic effects of Aβ (Rose et al., 2009). NPY did not only have an effect on offsetting the toxic effect of Aβ, but also restored the neurotrophin levels in neuroblastoma cells (Croce et al., 2011). Croce et al. (2013) pre-incubated primary rat cortical neurons with NPY and exposed them to Aβ25–35 fragments. They found that NPY mediated a decrease in miR-30a-5p expression and an increase in brain derived neurotrophic factor (BDNF) mRNA and protein levels, which possibly contributed to the neuroprotective effect of NPY in rat cortical neurons exposed to Aβ (Croce et al., 2013).

It is well known that excitotoxicity is a process of neuronal death caused by excessive or prolonged activation of glutamate (Glu) receptors (Lau and Tymianski, 2010). As a neurotransmitter, Glu is stored in vesicles within the presynaptic terminals. When nerve impulses stimulate the terminals to depolarize, voltage-dependent Ca^2+^ channels are activated, and extracellular Ca^2+^ flows into the presynaptic terminals to promote the release of Glu into the synaptic spaces in a Ca^2+^-dependent manner. Afterward, Glu combines to their receptors in the postsynaptic membrane, generating neuroexcitatory effects. The role of NPY in neuroprotection might be related to its ability to reduce Ca^2+^ influx in presynaptic nerve terminals. The ability of the Aβ peptide to form Ca^2+^-permeable pores in neuronal membranes is possibly related to the Aβ-initiated neuronal death. This is because Ca^2+^-permeable pores in neuronal membranes can induce excessive Ca^2+^ influx and neurotoxic cascades, which are responsible for neuronal death (Arai et al., 2019). Excessive Ca^2+^ influx could lead to mitochondrial function failure and finally result in neuronal death due to excitotoxicity (Connolly et al., 2014). Qian et al. (1997) reported that NPY reduces intracellular calcium concentrations via the inhibition of voltage dependent Ca^2+^ channels. It was also revealed that NPY has a neuroprotective effect that can reduce the excitotoxic role of glutamate, inhibit glutamate receptor overactivity, and rescue hippocampal, cortical, and retinal cells from necrosis or apoptosis via activation of Y2 and Y5 receptors. This neuroprotective effect of NPY was mediated by the activation of protein kinase A and p38K, which are key proteins in different intracellular pathways (Santos-Carvalho et al., 2013).

Neuronal loss is an important pathological feature of AD. Neuronal replacement therapies have already been reported for the treatment of neurodegenerative diseases (Bachoud-Lévi et al., 2006; Mendonça et al., 2015). Neurotransmitters and neuropeptides such as NPY can dynamically regulate adult neurogenesis (Lee et al., 2018). In this regard, Spencer et al. (2016) developed a lentiviral vector expressing NPY, which was fused to a brain transport peptide (apolipoprotein B) for widespread CNS delivery in an amyloid precursor protein-tg mouse model of AD in order to explore the function and role of NPY in neurogenesis of AD (Spencer et al., 2016). The results showed that the proliferation of neural precursor cells in the sub-granular zone of the hippocampus increased significantly without further differentiation into neurons. NPY regulated neurogenesis in the dentate gyrus, caudal subventricular zone (cSVZ), and subcallosal zone via the proliferative effect of Y1 receptors on neuroblasts (Howell et al., 2005; Thiriet et al., 2011). NPY promotes SVZ neurogenesis and increases the number of functional SVZ neurons through the Y1 receptors (Agasse et al., 2008). The neuronal proliferative effect of NPY is mediated by Y1 receptors, and further downstream through a kinase cascade involving protein kinase C and extracellular signal-regulated protein kinases 1 and 2 (Lecat et al., 2015). In the study by Agasse et al. (2008), stress-activated protein kinase/c-Jun N-terminal kinase (P-SAPK/JNK) was found in the cytoplasm and neurite-like structures colocalizing with tau, a microtubule-associated protein mainly present in axons, 6 h after treatment with NPY. Additionally, treatment with NPY increased the total length and number of P-SAPK/JNK-positive ramifications. These data suggest that NPY promotes axonal sprouting and neuronal differentiation through the activation of the SAPK/JNK pathway (Agasse et al., 2008). All the evidence indicate that NPY plays a role in AD by modulating neurogenesis.

Immune response also plays an important role in the pathogenesis of AD. Microglia are resident innate immune cells in the brain, and they play a crucial role in AD progression. Thus, overactivation and dysregulation of microglia might result in severe and progressive neurotoxicity (Hansen et al., 2018). Activation of Y1 receptors inhibited microglial cell activation (Thorsell and Mathe, 2017), while Y2 receptors had a protective effect against methamphetamine (METH)-induced cell death and microgliosis (Goncalves et al., 2012). It was reported that NPY can suppress neuroinflammatory responses and neurodegeneration by delivering NPY- apolipoprotein B to the brain, resulting in widespread reduction in astrogliosis. Therefore, NPY affects the attenuation of neuroinflammation by activating NPY receptor 1 and receptor 2 signaling pathways of microglia (Spencer et al., 2016). In general, NPY may play a therapeutic role in AD by modulating neurogenesis and neurotrophins, decreasing excitotoxicity, regulating calcium homeostasis, and attenuating neuroinflammation.

### NPY in Parkinson’s Disease

Parkinson’s disease is the second most common neurodegenerative disease. The most important pathological features of PD are selective degeneration and loss of dopaminergic neurons in the substantia nigra and the presence of Lewy bodies, primarily composed of fibrillar α-synuclein in the surviving neurons (Trigo-Damas et al., 2018). The etiology and pathogenesis of PD remain unclear, but are generally believed to be associated with genetic and environmental factors and aging. In Kerkerian et al. (1986), showed that the loss of the nigrostriatal dopamine pathway led to a significant increase in the number of NPY-expressing cells in the striatum in animal models of PD. Using *in situ* hybridization, it was shown that the expression of NPY mRNA in the caudate nucleus, putamen, and nucleus accumbens in patients with PD was markedly increased compared to that in healthy individuals (Cannizzaro et al., 2003). These suggest that NPY may be involved in the pathogenesis of PD.

In order to elucidate the role of NPY in PD, Decressac et al. (2012) investigated the survival of dopaminergic cells both *in vitro* and *in vivo* with animal models of PD. The study found that NPY exerted neuroprotective effect against 6-hydroxydopamine (6-OHDA)-induced toxicity both on nigral dopamine cell bodies and striatal terminals (Decressac et al., 2012). The neuroprotection of NPY is preferentially mediated via the Y2 receptors and may involve the activation of mitogen-activated protein kinase (MAPK) and Akt pathways (Decressac et al., 2012). It was shown that NPY interacted with different neurotransmitter systems and may play a role in the interaction between glutamate and dopamine-containing neurons (Cannizzaro et al., 2003). NPY protects dopamine neurons by inhibiting the release of glutamate in PD. Striatal cells are the targets of cortical glutamatergic neurons (Goto et al., 2013). Therefore, NPY could be a potential therapeutic target for PD by inhibiting the release of glutamate from the cortico-striatal neurons. A recent study suggests a direct effect of NPY on neuroglial elements. Using 6-OHDA-lesioned rats as a PD experimental model, the neuroprotective effect of NPY on microglia caused inflammation was identified through the specific binding of a ligand on the receptor translocator protein localized on microglial elements in the striatum and substantia nigra (Pain et al., 2019). Further studies from different perspectives are needed to elucidate the potential use of NPY as a therapeutic target in PD.

### NPY in Huntington’s Disease

Huntington’s disease is an autosomal dominant inherited degenerative disease of the nervous system, specifically the lesions in the cerebral cortex and striatum. HD is one of the polyglutamine disorders caused by an abnormal repetition of the GAG trinucleotide repeats (36 to 121 copies) within the coding sequence of the IT15 gene, which leads to the expansion of the polyglutamine chain in the huntingtin protein (Sameni et al., 2018). There is no effective way to prevent or palliate the disease of HD. The pathological process of HD can lead to encephalatrophy, especially causing damage in the striatum (Nopoulos, 2016). NPY is expressed by medium-sized GABAergic neurons in the striatum, which receives inputs from both cortical glutamatergic and nigral dopaminergic neurons and connects with neighboring cells. Dawbarn et al. (1985) measured NPY-LI both in HD brains and post-mortem human brains of a control group. The expression of NPY in HD was increased in the basal ganglia, cortex, and the subventricular zone (Dawbarn et al., 1985). In order to determine the mechanism underlying the function of NPY as a potential therapeutic target in tg mice model of HD, Decressac et al. (2010) further investigated the effects of a single intracerebroventricular (ICV) injection of NPY in a tg mouse model of HD (R6/2) by observing animal survival, body weight, changes in behavior, and pathology as well as adult neurogenesis (Decressac et al., 2010). They found that a single ICV injection of NPY in a tg mouse model of HD (R6/2) increased survival time and ameliorated the associated motoric and cognitive symptoms. Additionally, the number of newborn neuroblasts in the SVZ was enhanced compared with saline-treated animals. Thus, they proposed that ICV NPY promoted SVZ neurogenesis in wild-type mice. It was suggested that activated microglia contributed to the pathology of HD, and microglial activation was likely to increase over the course of the disease using [11C] (R)-PK11195 PET ([11C] raclopride positron emission computed tomography, a marker for dopamine D2 receptor binding) as an *in vivo* marker for activated microglia (Hansen et al., 2018). The distribution of NPY in retinal and cortical macroglial as well as the levels of NPY and the number of Y1 receptors were increased upon microglial activation (Li et al., 2014; Thorsell and Mathe, 2017; Campos et al., 2018). Increasing evidence was found to support the role of NPY in modulating microglial inflammatory responses (Ferreira et al., 2011, 2012). Microglial can regulate rapid rearrangement of the actin cytoskeleton enabling the cells to phagocytose. NPY inhibits IL-1β-induced phagocytosis by binding to Y1 receptors, a process accompanied by p38 MAPK and HSP27 activation (Ferreira et al., 2011, 2012). In addition, NPY was also observed to protect hippocampal cells against METH-induced toxicity through the release of BDNF from microglia, which has been shown to attenuate neuroinflammation by reducing astrocytosis and pro-inflammatory cytokine production (Xapelli et al., 2008). It is well known that activated microglia can release a variety of mediators including cytotoxic substances such as nitric oxide (NO), oxygen radicals, and inflammatory factors. We thus hypothesized that the mechanisms underlying NPY-mediated anti-inflammatory effects in neurodegenerative disorders may involve suppression of excessive inflammatory response and/or activation of the BDNF signaling pathway. In addition to reducing neuroinflammation in AD and PD, as mentioned above, NPY is also involved in inhibiting glutamate release, which could reduce glutamate excitotoxicity in HD (Decressac and Barker, 2012).

### NPY in Machado-Joseph Disease

Machado-joseph disease or spinocerebellar ataxia type 3 is a rare and progressive autosomal dominant neurodegenerative disorder. MJD has similar features to other polyglutamine diseases such as HD (de Assis et al., 2017). The pathogenesis of MJD involves CAG triplet expansion in exon 10 of the ATXN3 gene. The expanded polyglutamine stretch in mutant ataxin-3 protein leads to a gain of toxic function that eventually causes neurodegeneration predominantly in oculomotor, cerebellar, pyramidal, extrapyramidal, and peripheral motor systems (Toonen et al., 2017). Duarte-Neves et al. (2015) administered stereotaxic injection of adeno-associated viral vectors encoding NPY to obtain overexpression of NPY into the striatum and cerebellum of two different MJD mouse models. Their results demonstrated that NPY overexpression alleviated motor-coordination and balance disabilities, prevented mutant ataxin-3-induced increase in microglial immune reactivity, up-regulated BDNF levels, and reduced IL-6 mRNA levels in MJD mouse models (Duarte-Neves et al., 2015). Increased levels of BDNF and reduction of neuroinflammation are implicated as the beneficial effects of NPY on MJD (Duarte-Neves et al., 2015). The mechanisms underlying these effects need to be further investigated.

In summary, NPY modulates neurogenesis and neurotrophins, increases trophic support, decreases excitotoxicity, regulates calcium homeostasis, and attenuates neuroinflammation. Additionally, NPY also influences the common clinical manifestations of neurodegenerative diseases, such as depression and weight change (Cassie et al., 2017; Brown et al., 2018). Alterations in neurogenesis and neurotrophic factor expression or neuroinflammation, impairments in the serotonergic and noradrenergic systems, and hypothalamic-pituitary-adrenal (HPA) axis dysfunction, all contribute to the depressive symptoms of neurodegenerative disorders (Ayuob et al., 2018). NPY stimulated neurogenesis, increased BDNF levels, promoted the survival of newborn neurons, counteracted neuroinflammation, inhibited the release of pro-inflammatory cytokines, and attenuated the toxic effects of activated microglia (Decressac et al., 2011; Duarte-Neves et al., 2015). NPY also played a role in increasing the levels of serotonin and norepinephrine, decreasing HPA axis hyperactivity, and reducing plasma adrenocorticotropic hormone and cortisol plasma levels (Painsipp et al., 2008; Bahry et al., 2017).

**FIGURE 1:**
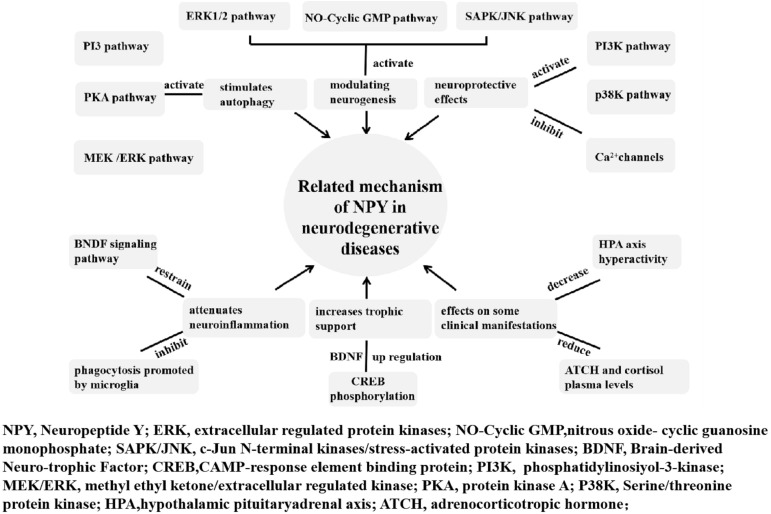
Related mechanism of NPY in neurodegenerative diseases. The neuroprotective and anti-neuroinflammatory roles of NPY in neurodegenerative diseases include modulating neurogenesis, increasing trophic support, exerting neuroprotective effects, affecting some clinical manifestations, attenuating neuroinflammation, and stimulating autophagy.

Weight loss occurs several years before diagnosis and is correlated with the severity and stage of diseases (Saute et al., 2012; Soto et al., 2012). Olfactory dysfunction is a clinical symptom of PD as well as a side effect of acetylcholinesterase inhibitors and dopaminergic drugs. It usually occurs early in the course of neurodegenerative diseases and results in inappetence eventually (Seo et al., 2018). Higher energy expenditure in PD, HD, and MJD results from dyskinesia and dystonia. Both increased energy consumption and reduced calorie intake may contribute to weight loss. NPY plays a vital role in the regulation of body weight and physiological control of food intake (Zhang et al., 2019). It is believed that NPY can be a potential therapeutic target in neurodegenerative diseases. The related mechanisms of NPY in neurodegenerative disease are summarized in Figure 1.

## Role of Npy in Neuroimmune Diseases

Neuroimmune diseases are a type of autoimmune disease related to hereditary susceptibility, and environmental and various stress factors. Neuroimmune diseases can be divided into CNS and peripheral nervous system (PNS) diseases. Multiple sclerosis (MS) is the most common type of CNS neuroimmune diseases, and Guillain-Barré syndrome (GBS) is the most common type of PNS neuroimmune diseases.

### NPY in Multiple Sclerosis

Multiple sclerosis is a chronic autoimmune disease of the CNS characterized by multifocal inflammation in the brain, extensive demyelination, axonal loss, and gliosis. Although, the exact pathogenesis of MS remains to be completely elucidated, CD4^+^T cell-mediated autoimmunity has been accepted as one of the most important aspects of MS pathogenesis (Lazibat et al., 2018). Microglia are resident immune cells in the CNS. In MS microglial are not only involved in CNS damage caused by immune response, but also play an important role in disease recovery and nerve regeneration (Zrzavy et al., 2017). NPY, through its Y1 receptor, can inhibit microglial activation, IL-Iβ production, as well as subsequent NF-κB-iNOS signal transduction, decrease the production of NO (Sørensen et al., 2012). NPY can also reduce the migration and phagocytosis of microglia by limiting the activity of microglia and avoiding excessive release of NO, glutamate, cytokines, and other cytotoxic substances from microglia. This effect is achieved by affecting the p38 signal system. It is also associated with heat shock protein 27 (Ferreira et al., 2012). Remarkably, NPY can inhibit the secretion of IFN-γ and enhance IL-4 secretion of murine lymphocytes, indicating that NPY shifts the help T cell 1 (Th)1/Th2 balance toward the Th2 phenotype (Levite, 2008). It was found that the levels of NPY were decreased in the cerebrospinal fluid of MS patients (Maeda et al., 1994).

Experimental autoimmune encephalomyelitis (EAE), an animal model of MS, is an inflammatory autoimmune disease which affects the CNS and is induced by myelin self-antigens (Chen Y. et al., 2019). The suppressive role of exogenous NPY has been demonstrated in EAE mice (Schmitz et al., 2017). Furthermore, Bedoui et al. (2004) tested whether repetitive administrations of NPY would exert any impact on EAE. They found that NPY can ameliorate symptoms and disease severity of EAE in a dose-dependent manner (Schmitz et al., 2017). This effect of NPY presumably occurs by decreasing IFN-γ secretion from autoreactive T lymphocytes and elevating the IgG1-IgG2a ratio of autoantigen-specific antibodies, which indicate that NPY favors Th2 response. When the Y1-receptor signaling was blocked immediately after immunization for EAE, an earlier onset of the disease was observed. This finding indicated that endogenous NPY plays a protective role in EAE induction and exerts its effects directly on T lymphocytes via the NPY Y1 receptor subtype (Schmitz et al., 2017).

The sympathetic nervous system (SNS) is mechanically and functionally affected in both rheumatoid arthritis and MS (Sternberg, 2012). Clinical studies also suggested that the defective crosstalk between SNS and the immune system might further precipitate the manifestations of MS because of considerable SNS dysfunction in patients with MS (Shahabi et al., 2006). Substantial evidence indicated that stress can precipitate or worsen symptoms of inflammation in MS. NPY significantly promoted stress coping and resiliency (Wagner et al., 2016). Neuropeptides secreted under stress could activate microglia and mast cells to release inflammatory molecules. This results in the maturation and activation of Th17 autoimmune cells, destruction of the blood brain barrier (BBB), and T cells entering the CNS, which can promote brain inflammation and cause MS (Karagkouni et al., 2013). These indications give us clues to further investigate the role of NPY in regulating autoimmune processes and to identify a new therapeutic target of MS/EAE.

### NPY in Guillain-Barré Syndrome

Guillain-Barré syndrome is a common acute immune-mediated inflammatory disease of the PNS characterized by inflammatory infiltration and damage to myelin sheaths and axons. Experimental autoimmune neuritis (EAN) is an animal model for studying the pathogenesis and treatment of GBS. Many studies have confirmed that the pathogenesis and progression of GBS/EAN involve a variety of immune cell subsets and a complex network of cytokines. Th1, Th2, Th17, and Treg cells, the four common subsets of CD4+T cells, restrict or antagonize each other by releasing their effector cytokines. The net effects of Th cytokines determine the direction of immune responses and the consequence of GBS/EAN (Nyati et al., 2011; Zhang et al., 2013). A Th1 oriented response, which involves an increase in the production of Th1 cytokines, including IFN-γ, TNF-α, IL-1β, and IL-6, is associated with a GBS acute-phase reponse to the immune response after infection. During the recovery phase, the levels of IFN-γ were decreased and the IL-4 was increased, indicating that a Th2 response is related to recovery from the disease (Gigi et al., 2008). Th17 cells, which are independent of Th1 cells, have been reported to play an important role in the progression of the disease in humans and animal models. The proportion of Th17 cells in the peripheral blood and the levels of IL-17A in the plasma of patients with GBS were increased during the acute phase. Moreover, the levels of IL-17A are correlated with the GBS disability scale score (Li et al., 2012). Tregs, a subset of CD4+T cells which play a crucial role in the maintenance of immune tolerance and prevention of autoimmunity, are significantly reduced in GBS patients and EAN animals (Harness and McCombe, 2008). Macrophage-mediated segmental demyelination is the pathological hallmark of GBS/EAN. The pivotal role of macrophages in nerve damage during GBS/EAN involves the direct phagocytotic attack on myelin and the secretion of several pro-inflammatory cytokines, including IL-1, IL-6, IL-12, and TNF-α (Shen et al., 2018). Macrophages have been primarily divided into two distinct subsets: pro-inflammatory macrophages (M1) and anti-inflammatory macrophages (M2). M1 macrophages are involved in inflammatory impairments of the myelin sheath via the release of pro-inflammatory Th1 cytokines such as IL-12 during the early course of GBS (Labonte et al., 2014). In contrast, M2 macrophages are associated with recovery from the disease through the secretion of anti-inflammatory cytokines and the facilitation of Th2 immune responses in the later stage of GBS (Shen et al., 2018). These cells interact and promote each other, and even regulate the immune effect directly. The functions and interactions of these cells are mainly achieved through the secretion of cytokines. These cytokines are interconnected, complex, and pleiotropic, and constitute a complex immune network contributing to the pathogenesis of GBS/EAN (Zhang et al., 2013).

**FIGURE 2:**
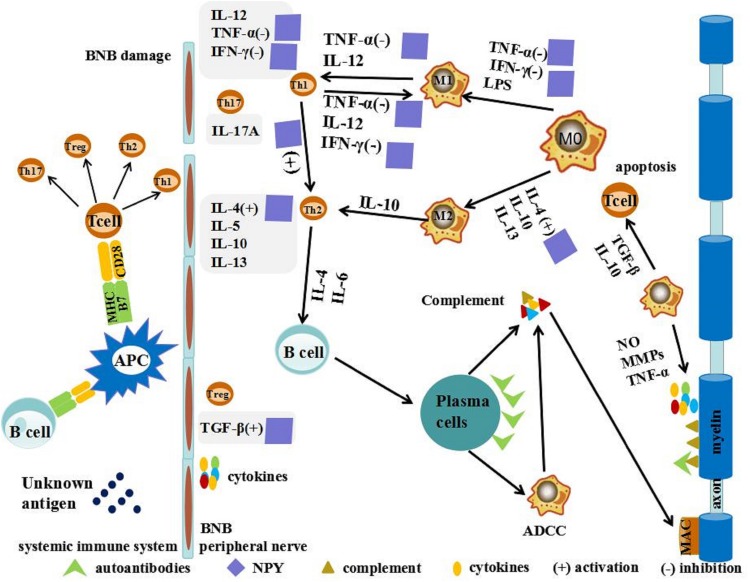
Possible action of NPY in GBS/EAN. T cells are activated by unknown antigens on antigen-presenting cells (APCs) through a combination of major histocompatibility complex (MHC), T cell receptor (TCR), and co-stimulatory signals in the systemic immune system. These activated neurogenic T cells differentiate into pro-inflammatory T helper cells (Th1, Th2, and Th17) and regulatory cell (Treg). Th1 secretes pro-inflammatory cytokines such as tumor necrosis factor (TNF)-α and interferon (IFN)-γ and interleukin (IL)-12 to activate macrophages (MΦ). MΦ have been primarily divided into two distinct subsets: pro-inflammatory macrophages (M1) and anti-inflammatory macrophages (M2). M1 macrophages promote breakdown of the blood-nerve barrier (BNB) by releasing nitric oxide (NO), matrix metalloproteases (MMPs), and TNF-α. M2 macrophages promote remyelination and tissue repair by secreting anti-inflammatory cytokines such as IL-10 and tumor growth factor (TGF-β) and promoting T-cell apoptosis. NPY shifts the Th1/Th2 balance toward the Th2 phenotype, activating secretion of IL-4 and TGF-β, and inhibiting secretion of IFN-γ and TNF-α.

Neuropeptide Y is capable of shifting Th1/Th2 balance toward the Th2 phenotype by inhibiting the secretion of IFN-γ and enhancing IL-4 secretion of murine lymphocytes (Levite, 2008). The action of NPY on the immune or inflammatory responses is mediated by Th17 cells, which seems to be critical in the initiation and perpetuation of autoimmune responses, facilitating the entry of autoreactive Th1 cells into the target tissue (Gonzalez-Rey et al., 2010). The role of NPY in down regulating the production of a wide spectrum of chemokines may be either a consequence of reducing infiltration of immune cells or a direct effect on Th17 differentiation and/or migration. This role of NPY in GBS is still poorly understood and needs intensive investigation (Gonzalez-Rey et al., 2010). More importantly, it was shown that NPY can decrease the secretion of the pro-inflammatory cytokine TNF-α from macrophages following stimulation with lipopolysaccharide (Pendharkar et al., 2017). NPY can also increase the production of anti-inflammatory cytokines including transforming growth factor beta (Zhou et al., 2008). Thus, NPY influenced the adherence, migration, phagocytosis of monocytes/macrophages, and secretion of cytokines (Figure 2; Painsipp et al., 2010; Dimitrijevic and Stanojevic, 2013; Singer et al., 2013; Farzi et al., 2015). In addition, the peripheral nerve is not only a significant source of NPY release, but also heavily innervates peripheral immune organs. In some local tissues, “synapse-like” structures are formed between sympathetic nerve endings and macrophages (Iriki and Simon, 2012; Cruz-Topete and Cidlowski, 2015). The complex immune network formed in GBS/EAN provides a physiological and anatomical bases for NPY to regulate directly local inflammation and immune function. This might offer a theoretical framework to explore the role of NPY in the progression of GBS/EAN and the regulating role and the mechanism of the GBS/EAN immune network. Thus, further research for new therapeutic targets of GBS/EAN is warranted.

## Prospects of Npy in Therapeutics

At present, in most animal models of neurodegenerative diseases, NPY exerts its protective effect through different mechanisms. NPY can modulate neurogenesis and neurotrophins, increase trophic support, decrease excitotoxicity, and regulate calcium homeostasis, attenuate neuroinflammation, as well as influence the clinical manifestations of neurodegenerative diseases. With extensive research and the deepening of our understating of NPY, its critical role in neuroimmune diseases has been gradually established. In the immune system, NPY is known to modulate immune cell trafficking, T helper cell differentiation, cytokine secretion, natural killer cell activity, phagocytosis, and the production of reactive oxygen species (Farzi et al., 2015). Besides directly affecting immune cells, NPY also acts as a paracrine and autocrine immune mediator, since immune cells such as macrophages, dendritic cells, and lymphocytes can produce and release NPY (Farzi et al., 2015). Based on the regulatory role of NPY in inflammatory immune response, we hypothesize the pathological mechanisms of NPY in neuroimmune diseases and its potential role as a therapeutic target in neurodegenerative and neuroimmune diseases.

There are a number of limitations of NPY as a therapeutic target, including a short half-life, the lack of channel of NPY across the BBB, and the inhospitable environment of the gastric mucosa. Moreover, the most difficult obstacles to overcome is the lack of passage across the BBB. Although prior studies have administered NPY via intravenous infusions, it is unclear how much, if any NPY enters the CNS (Chapman et al., 2013). In order to overcome this barrier, considerable effort needs to focus on seeking a kind of non-peptide agonist that can selectively act on different receptors of the CNS to exert its biological effect and achieving targeted treatment. At present, several selective NPY receptor agonists are already widely used in research, and new promising small molecules that can act as NPY receptor agonists or antagonists have been developed (Duvall et al., 2019). In addition to basic research, the clinical value of NPY needs to be further explored. There is an urgent need to explore the molecular mechanism of protective effect of NPY in neurodegenerative and neuroimmune diseases, for example which type of NPY receptor is the most important in CNS and what role does NPY play in different parts of brain tissue? If NPY can be used as a target to develop new drugs for treatment of neurodegenerative and neuroimmune disorders, it will be a major breakthrough in clinical practice.

## Conclusion

Neuropeptide Y has a potential therapeutic role in the most prevalent neurodegenerative and neuroimmune diseases. NPY stimulates neuronal survival and neuroproliferation, attenuates neuroinflammation, suppresses excitotoxicity, induces autophagy, and counteracts depressive symptoms and weight loss present in neurodegenerative diseases. Additionally, based on the immune regulatory function of NPY, we hypothesize that NPY is a new therapeutic target in neuroimmune diseases. However, the roles of NPY in these diseases are more complex than suggested. Further studies are required to investigate the intricate role of NPY in the pathogenesis and treatment of neurodegenerative and neuroimmune diseases.

## Author Contributions

CL and XW carried out the literature review and drafted the manuscript. SL, YZ, and JZ helped to draft the manuscript. KL conceived, designed, and coordinated the study. All authors read and approved the final manuscript.

## Conflict of Interest Statement

The authors declare that the research was conducted in the absence of any commercial or financial relationships that could be construed as a potential conflict of interest.
